# Prevalence of Multidrug-Resistant CTX-M Extended Spectrum Beta-Lactamase-Producing *Escherichia coli* From Different Bovine Faeces in China

**DOI:** 10.3389/fvets.2022.738904

**Published:** 2022-08-01

**Authors:** Xiaojuan Wei, Weiwei Wang, Ningning Lu, Lingyu Wu, Zhen Dong, Bing Li, Xuzheng Zhou, Fusheng Cheng, Kairen Zhou, Haijian Cheng, Hongmei Shi, Jiyu Zhang

**Affiliations:** ^1^Lanzhou Institute of Husbandry and Pharmaceutical Sciences of Chinese Academy of Agricultural Science (CAAS), Lanzhou, China; ^2^Key Laboratory of New Animal Drug Project of Gansu Province, Lanzhou, China; ^3^Key Laboratory of Veterinary Pharmaceutical Development, Ministry of Agriculture, Lanzhou, China; ^4^Shandong Institute of Animal Science and Veterinary Medicine, Jinan, China; ^5^Gannan Tibetan Autonomous Prefecture Institute of Animal Husbandry Science, Gannan, China

**Keywords:** blaCTX-M, *E. coli*, feces, yak, beef cattle, dairy cattle

## Abstract

CTX-M extended spectrum beta-lactamase-producing *Escherichia coli* cause severe health hazards in livestock breeding. To date, little is known about antibiotic resistance differences among bacterial isolates from yaks, cows, and beef cattle; therefore, the aims of this study were to analyse the prevalence of CTX-M-producing *E. coli* in yak, beef cattle, and dairy cattle feces from different provinces in China. A total of 790 fecal samples from yaks, beef cattle, and dairy cows were used. Among all the samples, 523 non duplicate *E. coli* isolates were identified, and 29.6% of samples harbored CTX-M producers. The results showed that these *E. coli* strains harbored 15 clusters of CTX-M genes: CTX-M-79, CTX-M-55, CTX-M-15, CTX-M-14, CTX-M-28, CTX-M-179, CTX-M-65, CTX-M-24, CTX-M-27, CTX-M-102, CTX-M-105, CTX-M-173, CTX-M-238, CTX-M-196, and CTX-M-10. The dominant resistance genes were CTX-M-15, CTX-M-14, and CTX-M-55. Moreover, the distribution of CTX-M genes was related to geographical region. Based on the above findings, we reasoned that bovines are potential reservoirs of antibiotic resistance, and this problem should be given adequate attention.

## Introduction

Bacterial resistance has been reported worldwide and affects global public health. Among all kinds of bacterial resistance, the CTX-M extended spectrum beta-lactamase (ESBL) is the most dominant beta-lactam enzyme, and it is found mostly among the members of Enterobacteriaceae (1). In recent years, the CTX-M gene has been reported in different countries (2–5). The CTX-M β-lactamase family can be divided into five groups based on their amino acid identities (6): the CTX-M-1, CTX-M-2, CTX-M-8, CTX-M-9, and CTX-M-25 groups. Within each group, the differences in the sequence identities of the ESBLs are <10% (6, 7). The prevalence of ESBLs is variable in different geographical locations. In recent decades, the detection rate of ESBL Enterobacteriaceae has increased dramatically. Misuse of antibiotics in food animals may lead to the dissemination of resistance genes.

*Escherichia coli* is a normal commensal in animals, and the bovine manure is often used as a source of organic fertilizer for farmland. Organic manure treated in compost form is considered to have better biosecurity in organic farming than raw manure (8). Adequate temperatures are effective in reducing the number of harmful microorganisms that can survive (9). Sometimes inappropriate compost or raw manure discharged into the soil can lead to the drug-resistant bacteria entering the soil along with antibiotic residues (10, 11). Competition for nutrients from other microorganisms in the soil is a factor in the survival of *E. coli* (12). However, it has been documented that antibiotics found in feces are highly ecotoxic and can disrupt the balance of the ecosystem and inhibit biological treatment systems, thus perpetuating their presence in the environment as persistent and non-degradable pollution (13). Animals, humans and the environment are linked with each other in a shared ecology, and enough evidence is available regarding the existence of ESBL-producing bacteria in different ecological niches (14, 15). In this study, to explore the level of antibiotic-resistant *E. coli*, especially the production of CTX-M, and to provide useful information about the epidemiology of ESBLs, fecal samples from bovines (yaks, dairy cows, and beef cattle) from different areas were investigated.

## Materials and Methods

### Sampling

**Figure 1:**
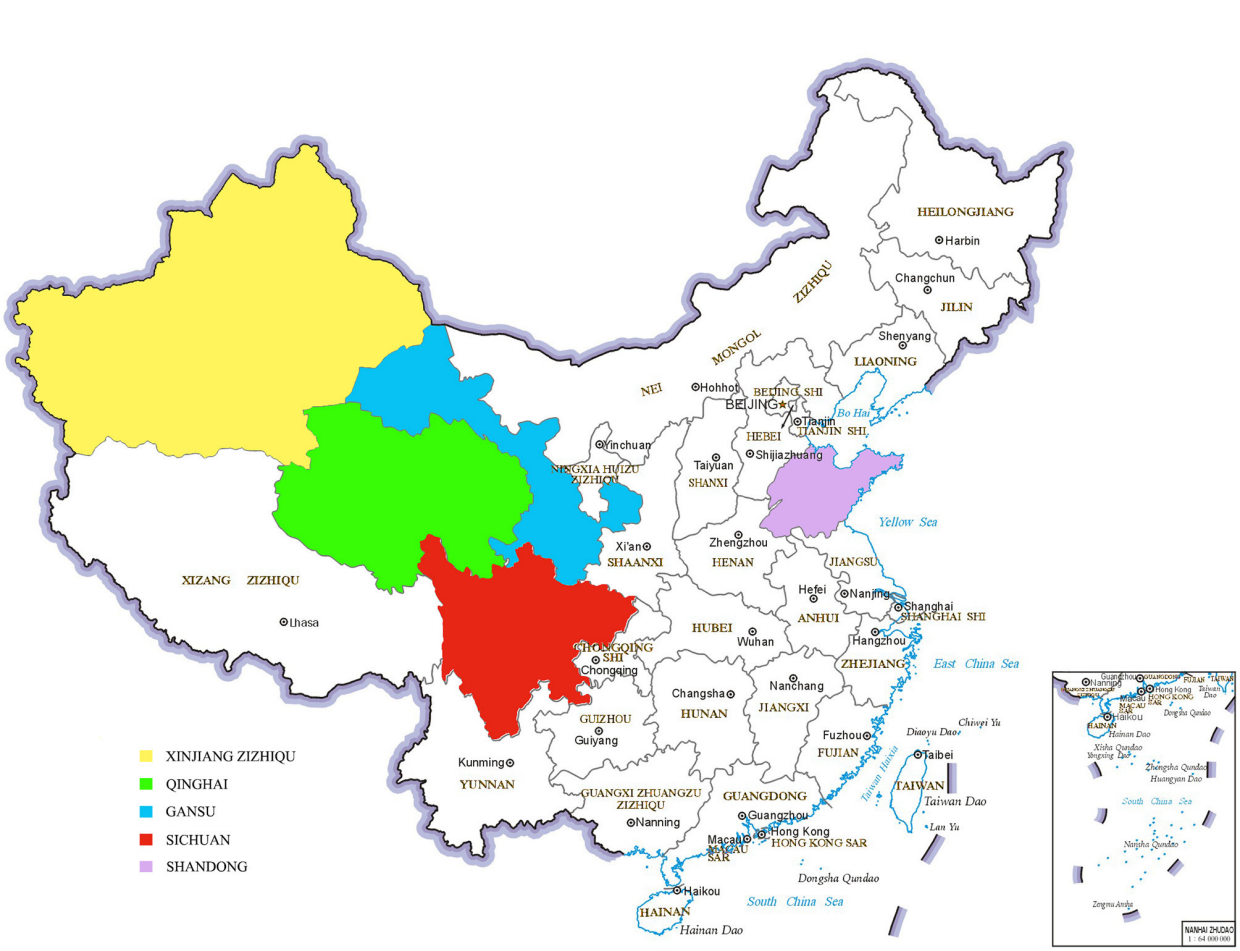
Map identification of sampling locations (Yellow is Xinjiang, green is Qinghai, blue is Gansu, red is Sichuan and purple is Shandong).

From 2018 to 2019, we collected 790 bovine fecal samples from Gansu, Qinghai, Sichuan, Shandong and Xinjiang in China (Figure 1). In the Gansu Province, we collected fecal samples from beef cattle (*n* = 180) and yaks (*n* = 90). In the Shandong Province, we collected fecal samples from beef cattle (*n* = 100) and dairy cattle (*n* = 80). In Xinjiang, we collected fecal samples from dairy cattle (*n* = 150). In Sichuan, we collected fecal samples from yaks (*n* = 100). In Qinghai, we collected fecal samples from yaks (*n* = 90). The fecal samples were collected after spontaneous defecation by sterile flocked swabs, stored in commercial universal transport medium (Yocon Biology Co., Beijing, China) and transported to the laboratory for analyses within 24 h of sampling.

### Bacterial Isolation and Identification

The isolation of *E. coli* was performed using selective chromogenic *E. coli* agar (Hopebio, Qingdao, China). In brief, samples were added to 2 ml of buffered peptone water and incubated at 35 ± 2°C for 16–18 h. Then, a loop of this broth was streaked onto chromogenic *E. coli* agar (Hopebio, Qingdao, China) and incubated at 35 ± 2°C for 18 h for *E. coli* selection. Presumptive *E. coli* colonies were identified by matrix-assisted laser desorption ionization–time of flight mass spectrometry (MALDI-TOF-MS) (Bruker Daltonics GmBH, Bremen, Germany). The data were processed by BioTyper 3.0 software. According to the manufacturer's recommendation, a score cut-off of ≥2.000 was considered *E. coli*. The identified *E. coli* isolates were cultured on commercial CHROMagar ESBL selective chromogenic medium (CHROMagar ESBL, France) and then the double-disc synergy test was used to detect the production of ESBL.

### Antibiotic Susceptibility Testing

The antibiotic susceptibility of the *E. coli* isolates from bovines was determined using a Kirby–Bauer disk diffusion approach (K-B programme) according to the recommendations of the Clinical and Laboratory Standards Institute (CLSI) (16) and the European Committee on Antimicrobial Susceptibility Testing (17). The following antibiotics were used: ampicillin (AMP), aztreonam (ATM), imipenem (IPM), cefuroxime (CXM), cefepime (FEP), ceftazidime (CAZ), cefmetazole (CMZ), cefotaxime (CTX), gentamicin (GEN), kanamycin (KAN), chloramphenicol (CHL), tetracyclines (TCY), streptomycin (STR), ciprofloxacin (CIP), and colistin (COL). The colistin results were evaluated according to the European Committee on Antimicrobial Susceptibility Testing (18). The results for the other antibiotics were evaluated according to the CLSI guidelines. *E. coli* ATCC 25,922 was used as a quality control strain. According to the results of the K-B disk assays, 48 multidrug-resistant *E. coli* isolates were selected to test the minimum inhibitory concentrations (MICs) by microdilution methods.

### Multilocus Sequence Typing

Conventional MLST was performed using seven housekeeping genes (*adk, fumC, gyrB, icd, mdh, purA* and *recA*), as described by Wirth et al. (19). The *E. coli* MLST database (https://pubmlst.org/bigsdb?db=pubmlst_escherichia_seqdef) was used to obtain the allele number and sequence type (ST).

### Detection and Sequencing of ESBLs (CTX-M, TEM, SHV), Carbapenemases (OXA, KPC, IMP, VIM), and MCR Genes

All isolates were screened for blaCTX-M genotypes by PCR. The primers for the CTX-M-1 group, CTX-M-2 group, CTX-M-8 group, and CTX-M-9 group, as well as the PCR conditions were as previously described (20). The primers for the CTX-M-25 group were as previously reported (21). The primers for *KPC* (22), *IMP* (23), *VIM* (24), *OXA* (25), *TEM* (26), *SHV* (26), and *MCR* were as previously described. Positive PCR products were further purified using a TIANgel purification kit (Tiangen, Beijing, China) and sent for sequencing. Sequencing was performed by the Tianqi Company (Gansu, China). The sequences obtained from the tests were entered into the BLAST database for comparison (27).

### Data Statistics and Analysis

The data involved in the study were kept in a special test logbook, which was then entered into Excel (Microsoft Corporation, Redmond, USA) for storage and processing. The results of the percentage comparisons were provided by the chi-square test in SPSS 26.0 (IBM, Armonk, USA).

## Results

### Bacterial Isolation and Identification

In total, 523 *E. coli* isolates were identified from 790 samples. The sampling area, species of animals and strain numbers of the *E. coli* isolates are reported in Table 1. Overall, 523 isolates were used in this study. Of these fifty-six strains of *E. coli* were obtained from yaks in Qinghai, 62 strains of *E. coli* were obtained from yaks in Sichuan, and 60 strains of *E. coli* were obtained from yaks in Gansu; 129 strains of *E. coli* were obtained from beef cattle in Gansu and 60 strains of *E. coli* were obtained from beef cattle in Shandong; and 108 strains of *E. coli* were obtained from dairy cows in Xinjiang and 48 strains of *E. coli* were obtained from dairy cows in Shandong.

**Table 1:** Sampling area, sources of species and strain number of *E. coli* isolates.

**Sampling area**	**Species sources**	**Strain number**
Xinjiang	Dairy cattle	108
Gansu	Beef cattle	129
	Yak	60
Qinghai	Yak	56
Sichuan	Yak	62
Shandong	Beef cattle	60
	Dairy cattle	48

### Antibiotic Susceptibility Testing

**Figure 2:**
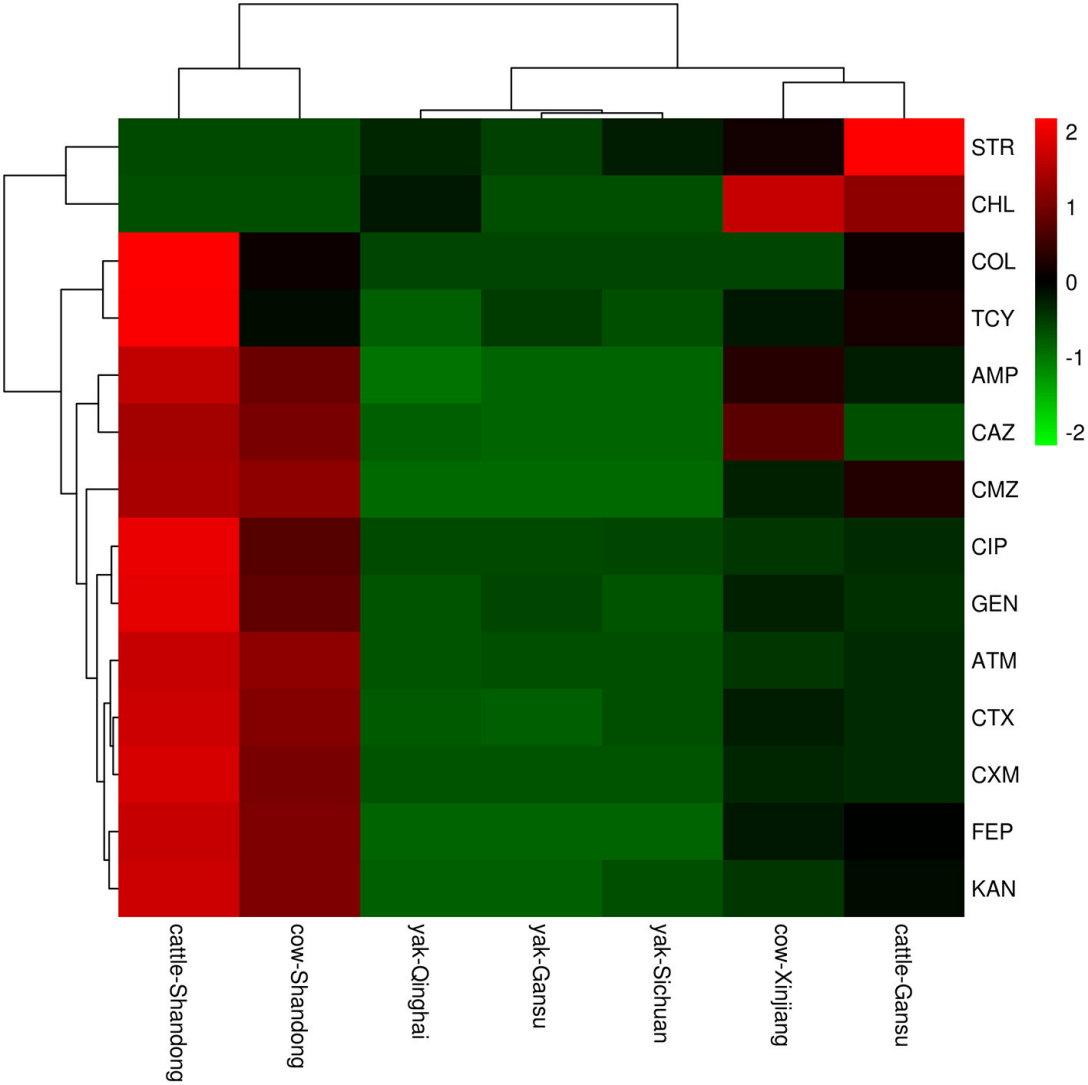
A heatmap of resistant phenotype percentage of *E.coli* isolation from different areas. The right side of the legend shows the color range of different R values. Species clustering trees are presented on the left side of the heat map.

In this study, 15 classes of antibiotics were used for sensitivity testing by K-B methods. The results (Figure 2) showed that the antimicrobial spectrum varied markedly between sampling areas; in addition, different animals in the same area had different antimicrobial profiles. In terms of animal species, antibiotic resistance rates in yaks were much lower than those in beef cattle and dairy cattle. Considering the sampling area, the highest antibiotic resistance was found in Shandong, followed by Gansu and Xinjiang, and the lowest resistance rates were found in Qinghai. Among the 15 antibiotics studied, the most frequently observed resistance phenotypes were ATM, CMZ, CAZ, CTX, AMP, and CIP.

**Figure 3:**
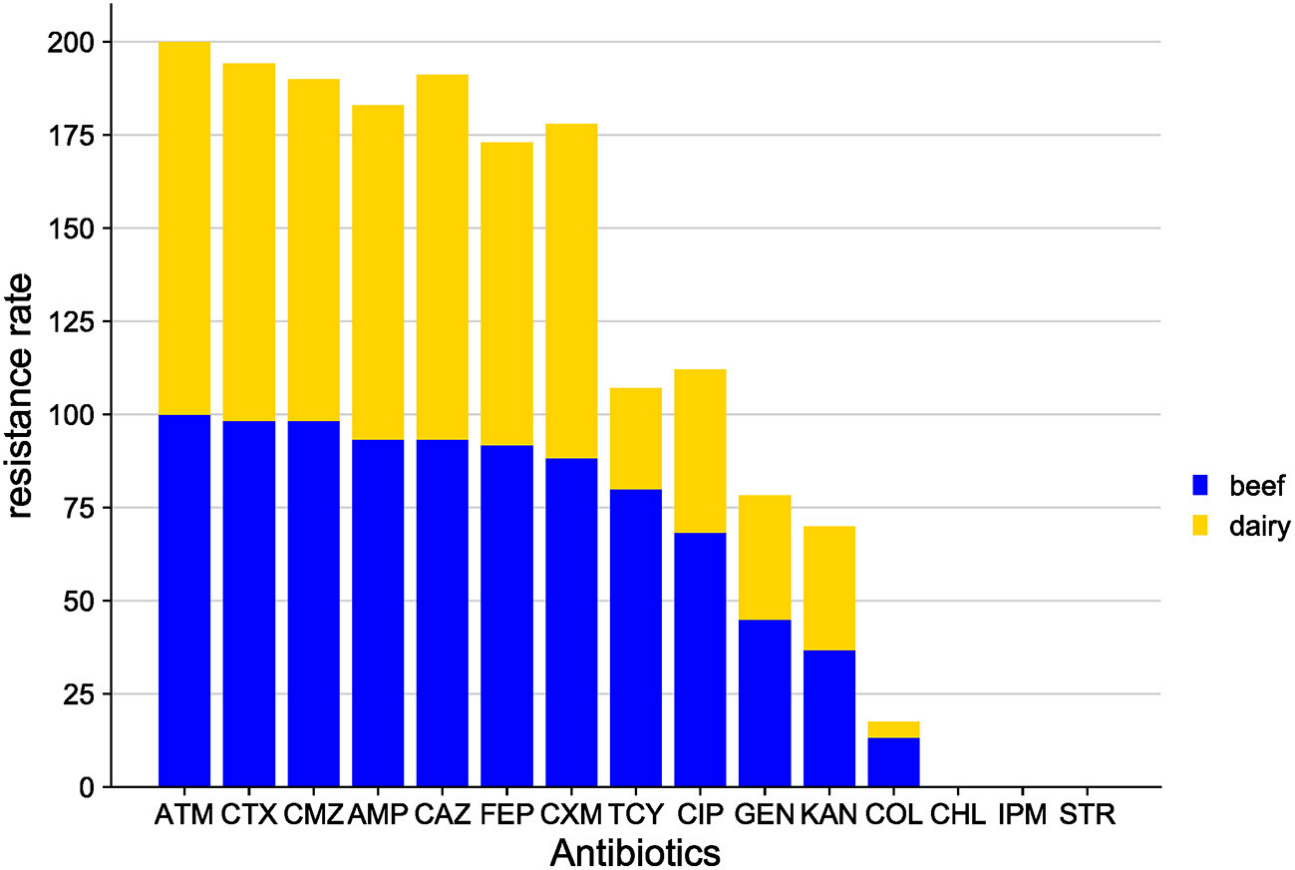
Distribution of antibiotic resistant phenotypes in *E. coli* from Shandong Province.

More specifically, the *E. coli* isolates from the Shandong samples were more resistant to the antibiotics tested than those from other areas. In *E. coli* from beef, resistance was found to the tested antibiotics, from highest to lowest, as follows: ATM (100.0%), CTX (98.3%), CMZ (98.3%), AMP (93.3%), CAZ (93.3%), FEP (91.7%), CXM (88.3%), TCY (80.0%), CIP (68.3%), GEN (45.0%), KAN (36.7%), and COL (13.3%). No *E. coli* isolates exhibited resistance to CHL, STR or IPM. In *E. coli* isolates from dairy cows, the highest resistance was to ATM (100.0%), followed by CAZ (97.9%), CTX (95.8%), CMZ (91.7%), AMP (89.6%), CXM (89.6%), FEP (81.3%), CIP (43.8%), KAN (33.3%), GEN (33.3%), TCY (27.1%), and COL (4.2%). No *E. coli* isolates were resistant to CHL, STR, or IPM (Figure 3).

**Figure 4:**
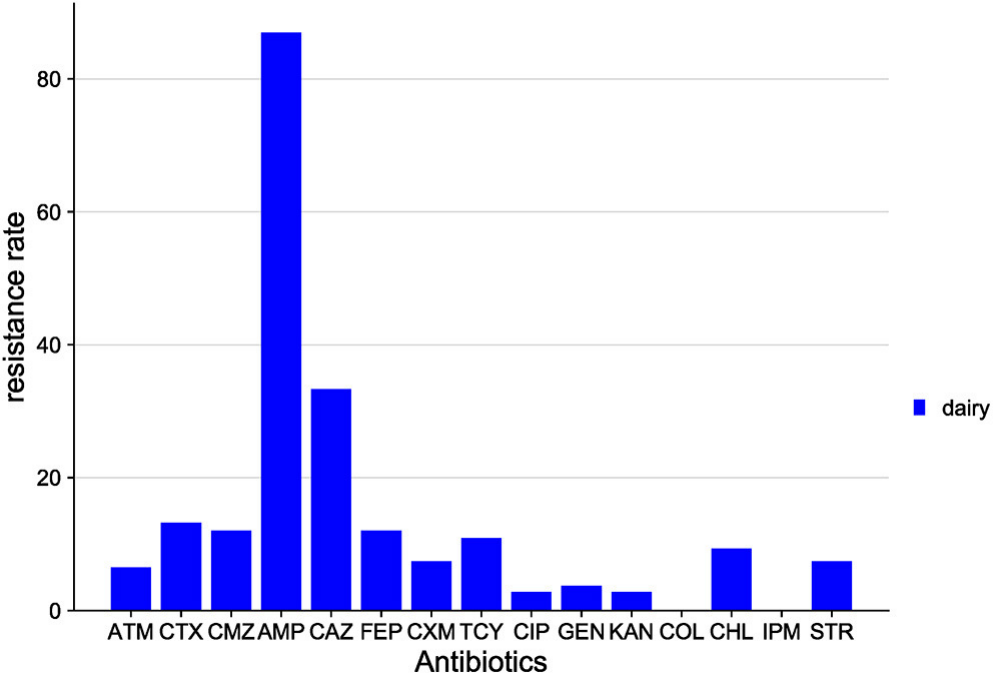
Distribution of antibiotic resistant phenotypes in *E. coli* from Xinjiang.

The distribution of drug resistance among *E. coli* isolates from Xinjiang cows is shown in Figure 4. The highest resistance was to AMP (87.0%), followed by CAZ (33.3%), CTX (13.2%), FEP (12.0%), CMZ (12.0%), TCY (10.9%), and CHL (9.3%). All isolates were sensitive to COL and IPM.

**Figure 5:**
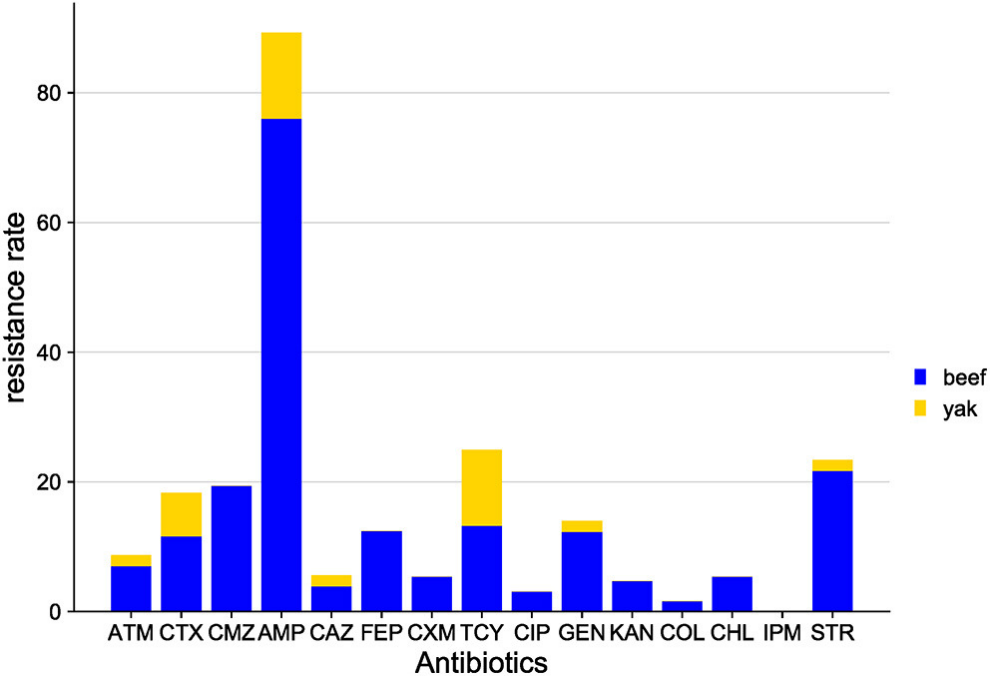
Distribution of antibiotic resistant phenotypes in *E. coli* from Gansu.

The distribution of antibiotic resistance among *E. coli* isolates from Gansu samples is shown in Figure 5. The antibiotic sensitivity of *E. coli* isolates from beef cattle and yaks was different. Among the beef cattle samples, of the 15 antibiotics tested, *E. coli* isolates presented resistance to 14 antibiotics, while *E. coli* isolates from yaks were only resistant to 7 antibiotics. Among the beef cattle samples, the highest resistance was found to AMP (76.0%), followed by STR (21.7%), while the lowest resistance was found to IPM (0%). Among yak samples, the highest resistance was to AMP (13.3%), followed by CTX (6.7%) and TCY (6.7%), while yak *E. coli* isolates were sensitive to 9 antibiotics (FEP, CMZ, COL, IPM, CXM, CIP, KAN, and CHL).

**Figure 6:**
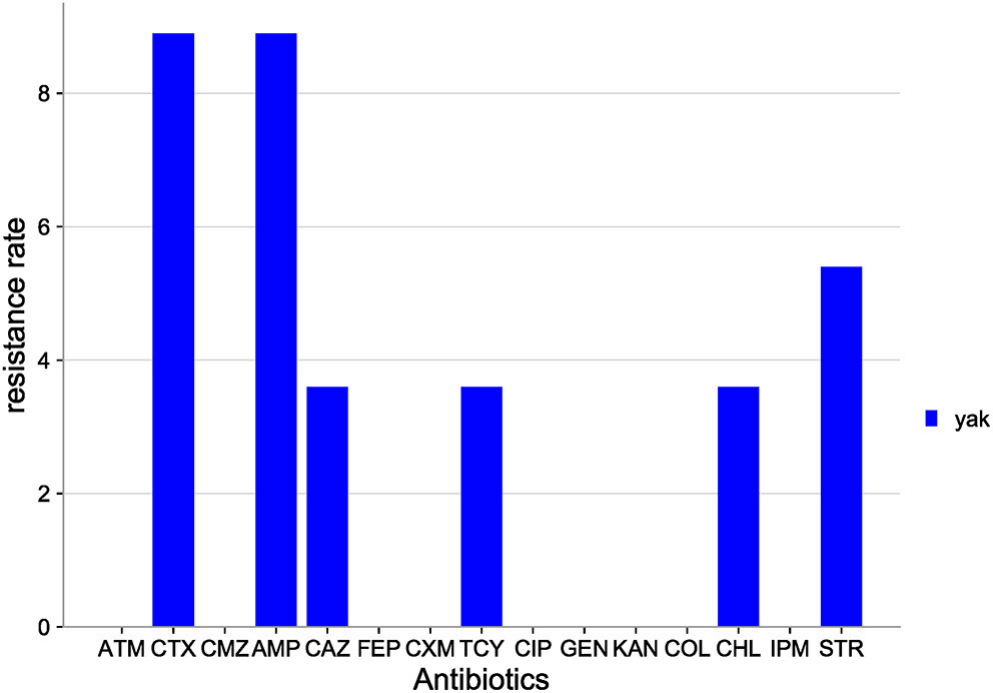
Distribution of antibiotic resistant phenotypes in *E. coli* from Qinghai.

The distribution of antibiotic resistance among *E. coli* isolates from Qinghai yak is shown in Figure 6. Among the 15 antibiotics tested, *E. coli* isolates were resistant to six antibiotics: AMP (8.9%), CTX (8.9%), STR (5.4%), TCY (3.6%), CHL (3.6%), and CMZ (3.6%). *E. coli* isolates were sensitive to the rest of the antibiotics tested (FEP, CMZ, COL, IPM, ATM, CXM, CIP, GEN, and KAN).

**Figure 7:**
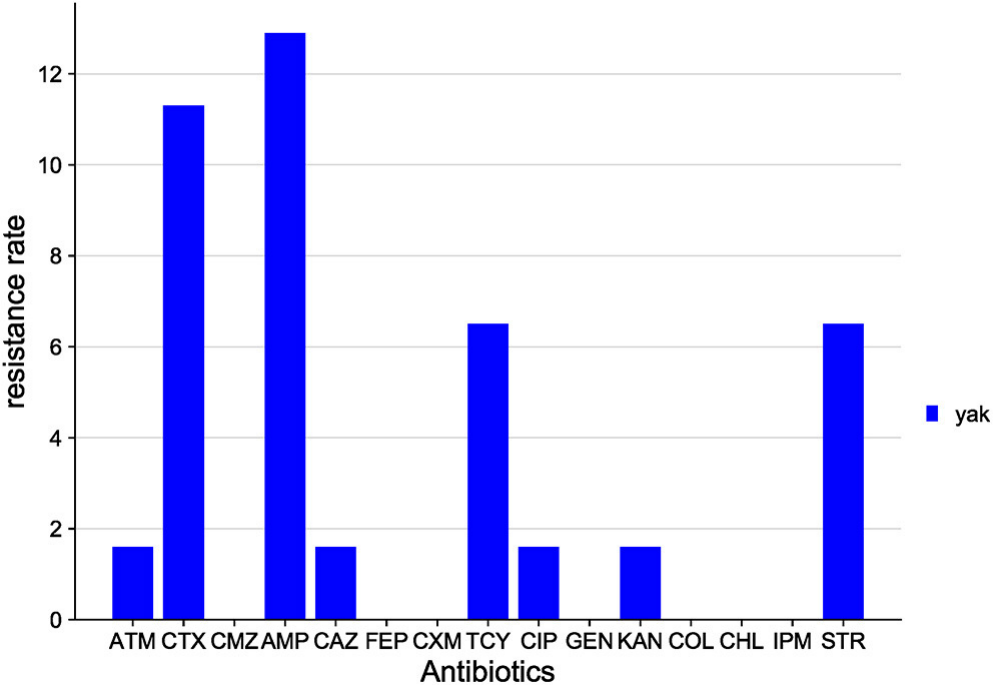
Distribution of antibiotic resistant phenotypes in *E. coli* from Sichuan.

The distribution of antibiotic resistance among *E. coli* isolates from the Sichuan yak is shown in Figure 7. Among the 15 antibiotics tested, *E. coli* isolates were resistant to 8 classes: AMP (12.9%), CTX (11.3%), STR (6.5%), TCY (6.5%), ATM (1.6%), CIP (1.6%), KAN (1.6%), and CAZ (1.6%). *E. coli* isolates were sensitive to the rest of the antibiotics tested.

**Figure 8:**
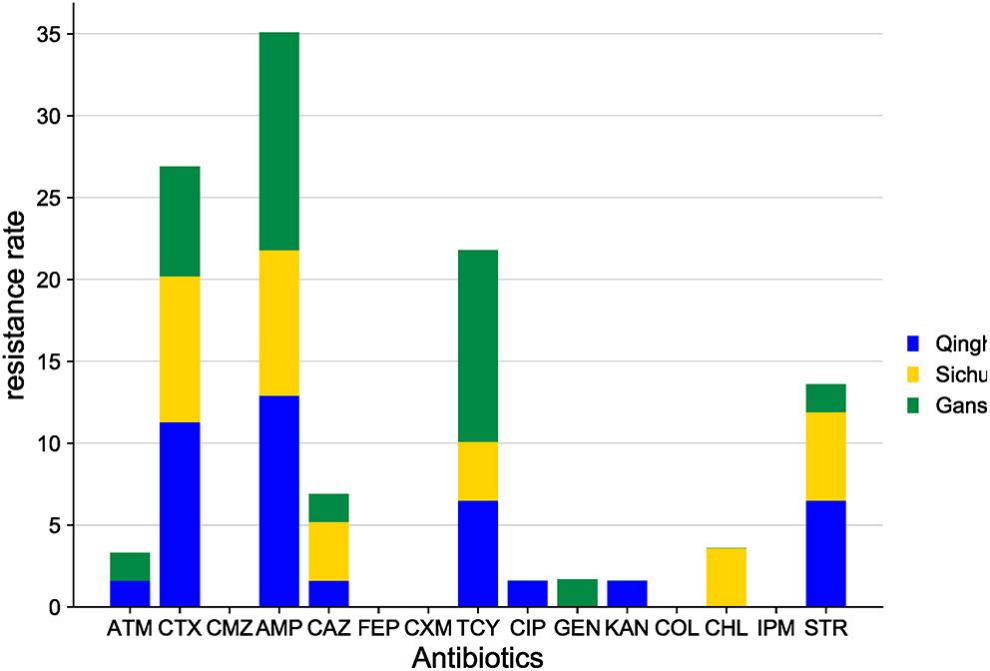
Comparison of antibiotic resistance in *E. coli* from yaks in different areas.

*E. coli* isolates from yaks share the characteristics of high resistance to AMP, CTX, TCY, STR, and CAZ (Figure 8) and sensitivity to CXM, FEP, CMZ, COL, and IPM.

Moreover, multidrug-resistance phenotypes (resistance to >2 of the antibiotic classes tested) were observed. The results are shown in Figure 2. *E. coli* isolates from Shandong were the most represented. The most prevalent multidrug resistance phenotypes were AMP-FEP-CMZ (*n* = 4) and CTX-FEP-CMZ (*n* = 3), found in the beef cattle from Gansu; AMP-CAZ (*n* = 10), CTX-CAZ (*n* = 5), and AMP-CAZ-CTX-FEP-CMZ (*n* = 4), found in dairy cattle from Xinjiang; AMP-FEP-ATM-CAZ-CMZ-CTX-IPM (*n* = 7), MP-CXM-FEP-CAZ-CMZ-ATM-CTX-IPM (*n* = 6), and GEN-AMP-CXM-CIP-ATM-FEP-CMZ-CTX-KAN-IPM (*n* = 4), found in dairy cattle from Shandong; and AMP-CXM-CIP-FEP-CAZ-TCR-CMZ-CTX-ATM-IPM (*n* = 8), and AMP-CXM-FEP-CAZ-TCR-CMZ-CTX-ATM-IPM (*n* = 7), found in beef cattle.

The MIC results of 48 selected multidrug-resistant *E. coli* isolates are shown in Table 2. The results were similar to those of the K-B methods.

**Table 2:** MIC of selected *E.coli* isolates.

**Bovine species**	**Area**	**Strains**	**MIC (mg/L)**													
			**CIP**	**CHL**	**AMP**	**COL**	**CTX**	**CAZ**	**FEP**	**KAN**	**GEN**	**IMP**	**ATM**	**CXM**	**CMZ**	**TCY**
Yak	Gansu	GN25	32	8	128	2	256	256	256	16	8	2	256	256	64	64
		GN16	16	16	256	1	256	256	256	64	8	2	256	256	256	256
		GN69	16	8	256	2	256	256	256	128	4	2	256	256	256	256
		GN12	32	8	128	2	256	256	256	16	2	2	256	256	64	64
		GN50	64	16	256	2	256	256	256	64	16	2	256	256	64	64
		GN9-1	32	16	128	1	64	256	256	256	32	2	256	256	64	64
	Sichuan	KD22-3	128	4	256	2	256	256	256	32	4	2	256	256	256	256
		KD2-2	4	2	256	2	256	256	256	8	4	1	256	256	256	16
		KD13-1	32	2	256	2	256	256	256	8	2	2	256	256	256	256
		KD2-3	64	8	256	1	256	256	256	64	2	2	256	256	128	128
Cow	Xinjiang	XJ11-2	32	64	256	0.25	256	256	256	32	256	1	128	256	64	256
		XJ20-3	256	256	256	0.5	256	256	256	512	256	2	256	128	256	256
		XJ5-1	8	64	256	2	256	256	256	16	4	2	256	256	256	256
		XJ6-2	128	256	256	2	256	256	256	256	8	2	256	256	64	64
		XJ24	32	128	256	2	256	256	256	256	16	2	256	256	256	256
		XJ29-1	16	64	256	2	256	256	256	256	4	2	256	256	128	64
		XJ36-2	64	128	256	2	256	128	256	32	16	2	256	256	256	256
		XJ46-2	128	128	256	2	256	256	256	32	16	2	256	256	64	64
		XJ51-2	8	4	256	2	256	256	256	8	2	2	256	32	64	64
		XJ55	8	64	256	2	256	256	256	128	256	2	256	16	256	256
		XJ64	32	128	64	1	64	128	256	256	4	2	256	8	16	64
	Shandong	GS01-3	2	16	8	1	256	256	2	8	0.25	1	128	256	64	16
		GS02-2	256	16	256	2	256	256	2	32	256	2	256	128	32	8
		GS03-4	256	2	512	2	128	256	1	256	1	2	256	256	256	2
		GS04-2	2	1	128	0.25	128	128	128	4	1	1	128	128	128	4
		GS05-2	256	8	256	2	256	256	256	32	256	2	256	256	256	4
		GS06-4	2	16	512	0.5	256	512	512	32	2	2	64	256	128	8
		GS07-2	256	16	256	0.5	128	256	256	256	16	2	256	128	256	16
		GS08-2	256	8	512	0.5	256	256	256	128	1	2	256	256	256	8
		GS010-5	64	0.5	128	0.25	128	128	128	64	256	0.5	128	128	256	
Beef	Gansu	ZYB8-1	256	128	256	2	512	256	256	256	8	2	256	256	128	128
		ZYB16-1	8	512	128	2	256	256	256	16	8	1	256	256	256	256
		ZYB29	256	512	256	1	256	256	256	256	32	2	256	256	256	256
		ZYB39	128	32	256	2	256	256	256	256	256	1	128	256	256	256
		ZYB58	256	128	128	2	256	256	256	32	8	2	256	256	64	64
		ZYB62	16	128	256	2	256	256	256	256	256	2	256	256	256	32
		ZYB67-1	128	256	256	2	256	256	256	128	8	2	256	256	128	64
		ZYB21	16	32	256	1	256	256	256	16	2	2	256	256	128	64
		ZYB42-3	16	8	256	2	128	512	256	8	4	2	256	256	256	64
		ZYA6-2	16	4	128	2	64	128	256	8	2	2	256	256	128	64
		ZYC58-2	8	512	128	2	64	128	128	128	8	2	256	256	128	64
	Shandong	YX012	256	128	256	2	256	256	256	256	128	2	512	128	512	128
		YX003	256	128	512	2	256	256	256	64	0.5	2	256	256	256	128
		YX013	128	16	512	1	32	64	32	8	256	2	128	32	64	64
		YX015	256	32	256	0.5	256	256	256	64	4	1	256	256	256	32
		YX016	512	512	512	2	128	512	512	512	256	1	128	512	512	32
		LC1138	64	512	256	0.5	256	256	256	8	1	1	256	256	128	4
		LC1010	1	128	256	0.5	256	256	256	32	1	2	256	256	256	16

### MLST

MLST analysis showed that 44 ST types were obtained from the 144 *E. coli* isolates, and they were further classified into 9 groups and 35 singletons. The ST types of the *E. coli* isolates were diverse; of them, the most predominant ST was ST95 (*n* = 9, 6.25%), followed by ST93 (*n* = 8, 5.6%), ST156 (*n* = 7, 4.9%), ST446 (*n* = 6, 4.2%), ST392 (*n* = 6, 4.2%), ST10 (*n* = 4, 2.8%), ST155 (*n* = 4, 2.8%), ST744 (*n* = 4, 2.8%), ST1637 (*n* = 4, 2.8%) and ST6345 (*n* = 4, 2.8%).

**Figure 9:**
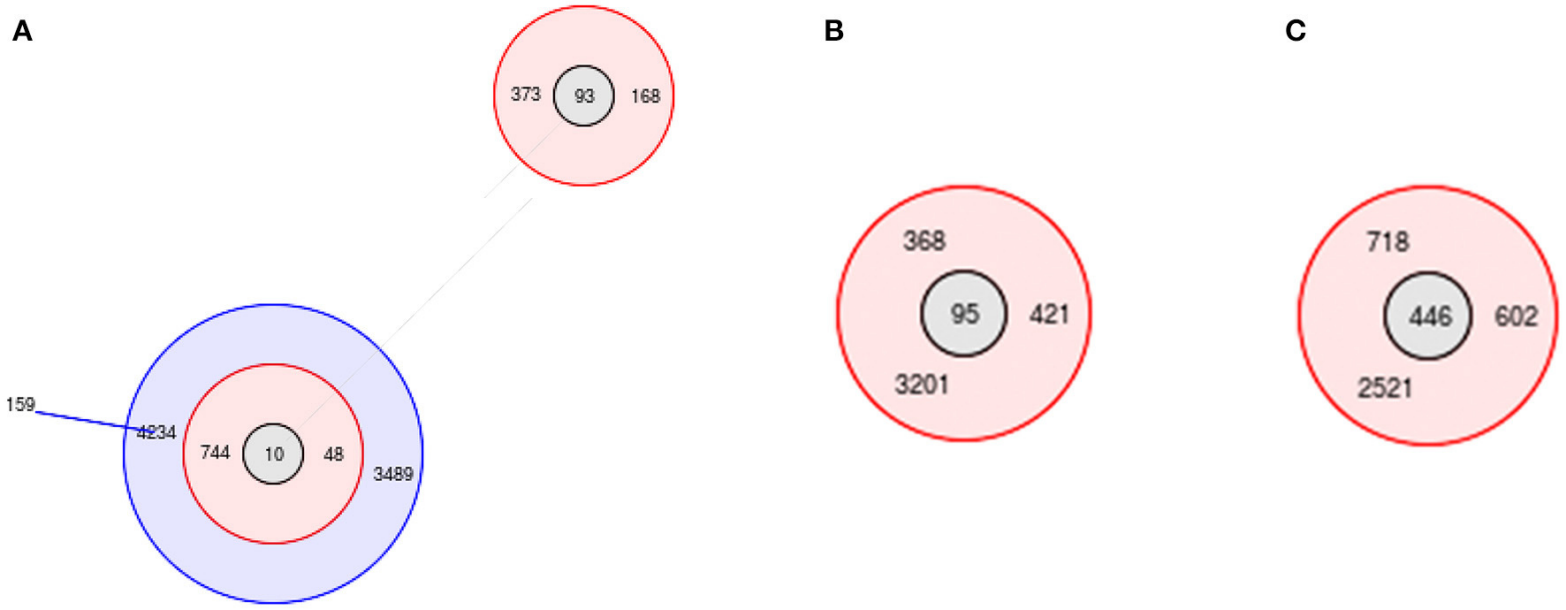
MLST profiles of *E.coli* isolates. **(A)** group 2; **(B)** group 4; **(C)** group 7.

Group 2 was centered on ST10, which was the largest, containing 9 ST types (Figure 9A); group 4 was centered on ST95, containing 4 ST types (Figure 9B); group 7 was centered on ST446, containing 4 ST types (Figure 9C); and the rest of the groups had no center. Group 1 contained 3 ST types, group 3 contained 4 ST types, group 5 contained 2 ST types, group 6 contained 4 ST types, group 8 contained 4 ST types, and group 9 contained 2 ST types.

Compared with ST10, only one allele of ST744 and ST48 was different, and two alleles of ST4234, ST3489, ST373, and ST168 were different. Among the 35 singletons, there were more than 5 alleles that were not identical to each other.

### PCR of Resistance Genes

We screened ESBL and carbapenemase genotypes by PCR in the *E. coli* isolates from yaks, beef cattle, and dairy cows. The results showed that the distribution of CTX-M genotypes was distinct in different bovines and in various sampling areas (Tables 3, 4). Considering the animal species, yaks had the fewest number of *E. coli* isolates harboring the CTX-M genotype (7.3%, 13/178); beef cattle had the largest number of *E. coli* isolates harboring the CTX-M genotype (50.8%, 96/189); and dairy cows had an intermediate number of *E. coli* isolates harboring the CTX-M genotype (30.8%, 48/156). Based on the geographical area, the highest prevalence of *E. coli* carrying the CTX-M gene was found among the Shandong samples (56.5%, 61/108), followed by the Gansu samples (31.7%, 60/189), the Xinjiang samples (22.2%, 24/108), the Sichuan samples (12.9%, 8/62), and finally, the lowest prevalence was among the Qinghai samples (3.6%, 2/56).

**Table 3:** The distribution of CTX-M genotypes in different bovines and sampling areas.

	**Geographical distribution**						
	**Shandong**		**Xinjiang**	**Gansu**		**Qinghai**	**Sichuan**
**Animal species**	**Beef cattle**	**Dairy cattle**	**Dairy cattle**	**Beef cattle**	**Yak**	**Yak**	**Yak**
CTX-M genotypes	CTX-M-15 (*n* = 24)	CTX-M-15 (*n* = 14)	CTX-M-14 (*n* = 5)	CTX-M-14 (*n* = 12)	CTX-M-65 (*n* = 1)	CTX-M-14 (*n* = 2)	CTX-M-14 (*n* = 3)
	CTX-M-238 (*n =* 12)	CTX-M-238 (*n =* 8)	CTX-M-65 (*n =* 3)	CTX-M-55 (*n =* 2)	CTX-M-65/14 (*n =* 1)		CTX-M-65 (*n =* 1)
	CTX-M-10 (*n =* 1)		CTX-M-14/24 (*n* = 1)	CTX-M-65 (*n* = 3)	CTX-M-79/55/15/238 (*n* = 1)		CTX-M-65/14 (*n* = 2)
	CTX-M-15/238 (*n =* 2)		CTX-M-79/55 (*n* = 4)	CTX-M-65/14 (*n* = 2)			CTX-M-79/55/15/238 (*n* = 1)
			CTX-M-79/55/65 (*n* = 1)	CTX-M-65/24 (*n* = 1)			CTX-M-79/55/14/27 (*n* = 1)
			CTX-M-79/55/28 (*n* = 1)	CTX-M-55/14 (*n* = 4)			
			CTX-M-79/55/14 (*n* = 1)	CTX-M-55/65 (*n* = 1)			
			CTX-M-14/24/65 (*n* = 1)	CTX-M-14/24 (*n* = 2)			
			CTX-M-14/27/105 (*n* = 1)	CTX-M-79/55 (*n* = 15)			
			CTX-M-14/27/102 (*n =* 1)	CTX-M-79/55/14 (*n =* 5)			
			CTX-M-79/55/15/238 (*n* = 2)	CTX-M-79/55/65 (*n* = 2)			
			CTX-M-79/55/14/65 (*n* = 1)	CTX-M-79/55/179 (*n* = 2)			
			CTX-M-79/55/14/179 (*n* = 1)	CTX-M-55/14/24 (*n* = 1)			
			CTX-M-55/65/14/24/196/173 (*n* = 1)	CTX-M-14/24/173 (*n* = 1)			
				CTX-M-79/55/14/65 (*n* = 2)			
				CTX-M-79/55/14/28 (*n* = 1)			
				CTX-M-79/55/14/24/65 (*n* = 1)			

**Table 4:** The distribution of TEM genotypes in different bovines and sampling areas.

	**Geographical distribution**						
	**Shandong**		**Xinjiang**	**Gansu**		**Qinghai**	**Sichuan**
**Animal species**	**Beef cattle**	**Dairy cattle**	**Dairy cattle**	**Beef cattle**	**Yak**	**Yak**	**Yak**
TEM genotypes	TEM-1 (*n* = 24)	TEM-1 (*n* = 12)	TEM-1 (*n* = 11)	TEM-1 (*n* = 33)	/	/	TEM-1 (*n* = 2)
	TEM (*n* = 6)	TEM (*n* = 7)	TEM (*n* = 8)	TEM (*n* = 18)	/	/	/

In yaks, the main genotype was CTX-M-14 (5.1%, 9/178). In beef cattle of Gansu, the most prevalent genotypes were CTX-M-55 (27.9%, 36/129), CTX-M-14 (24.0%, 31/129) and CTX-M-79 (21.7%, 28/129). In dairy cows from Xinjiang, the most common genotypes were CTX-M-14 (12.0%, 13/108), CTX-M-55 (11.1%, 12/108), and CTX-M-79 (10.2%, 11/108). In dairy cows and beef cattle from Shandong, the most prevalent genotypes were CTX-M-15 (37.0%, 40/108) and CTX-M-238 (20.4%, 22/108). CTX-M-79 was always present, accompanied by CTX-M-55.

In addition, many isolates carried more than one CTX-M gene. In *E. coli* isolates from Xinjiang, 4.6% (5/108) carried two CTX-M genes and 5.6% (6/108) carried three CTX-M genes. In *E. coli* isolates from beef cattle in Gansu, 19.4% (25/129) carried two CTX-M genes and 8.5% (11/129) carried three CTX-M genes. In *E. coli* isolates from Shandong, 1.9% (2/108) carried two CTX-M genes and no isolates carried three CTX-M genes.

This study showed that CTX-M genotypes were common in bovines, and the most prevalent were CTX-M-55, CTX-M-79, and CTX-M-15. Moreover, CTX-M-55 and CTX-M-15 emerged alone, while CTX-M-79 always emerged with CTX-M-55.

In *E. coli* isolates from Shandong, the detection of TEM was 45.4% (49/108), followed by isolates from Gansu beef at 39.5% (51/129), isolates from Xinjiang cows at 17.6% (19/108) and isolates from Sichuan at 3.2% (2/62). No IMP, VIM, OXA, SHV, KPC, or MCR were detected in any *E. coli* isolate.

## Discussion

In this study, fecal samples were collected from yaks, beef cattle, and dairy cattle from the five provinces in China to investigate the prevalence of the CTX-M resistance gene. The results showed that the most predominant resistance gene types were CTX-M-15, CTX-M-14, CTX-M-55, CTX-M-79, and TEM-1. This study suggests that bovines may be carriers of the CTX-M genotype. It is necessary to examine the factors that contribute to its dissemination (28).

Understanding antibiotic use is essential for drawing conclusions about the epidemiological links between antibiotic use in farm animals, antibiotic resistance, animal health, and human health. In China, the majority of cattle farming is located in the northern regions; based on this, the farming models in the central and eastern regions consist mainly of modern intensive farming, as well as a very small proportion of individual farms. In the west, however, while the dairy industry is also concentrated in intensive farming, beef cattle and the endemic breed yak are often farmed in a diversified manner. While common beef cattle are generally jointly owned by large farms and individual households, yaks are generally owned entirely by herders.

According to a survey, mastitis, uterine infections, and enteritis are often treated with antibiotics in dairy farms in China. In beef cattle farming, respiratory disease is often the top cause of morbidity, with hoof disease being a close second. This is similar to other parts of the world (29, 30). We know from the beef farm veterinarians that large amounts of ceftiofur and florfenicol are being used to control respiratory outbreaks following calf transfers and weather changes (31, 32). Ceftiofur with non-steroidal anti-inflammatory drugs (NSAIDs) is also frequently used due to the high incidence of diseases such as laminitis. According to previous literature, the most commonly used antibiotic in US cattle farms is penicillin, which accounts for 32% of the total antibiotic use, much higher than other antibiotics (33, 34). First-generation cephalosporins, penicillin, and third-generation cephalosporins are the most commonly used in Canadian dairy farms (29). Regarding the Australian region, antibiotic use on New Zealand cattle farms includes mainly penicillin, macrolides and cephalosporins (35). The most common drugs used on cattle farms in the UK are β-lactams, with third-generation cephalosporins, such as ceftiofur sodium, being the most common regardless of route of administration, followed by penicillin/streptomycin combinations (36). In other parts of Europe, it has been reported that Austria has a similar use profile (37). In addition, a pan-European antimicrobial susceptibility monitoring programme, the VetPath study, has shown that *E. coli* isolated in Europe is generally highly resistant to cephalosporins (38). This is similar to the situation on large intensive farms in Shandong, Xinjiang and Gansu. Our results showed that the samples collected from Shandong had the most complicated drug-resistance phenotypes, and multidrug resistance was the most serious in this region. The yak samples had the least complicated resistance profiles. Among the antibiotics tested, it is worth noting that the detection rate for colistin resistance was low. This is because colistin was banned as a feed additive by the Chinese government in 2017 but can still be used therapeutically (39). It is also interesting to note that the detection rate for imipenem- and aztreonam-resistant *E. coli* was very high in Shandong, but these two types of drugs are not used in the farming process. We hypothesize that this is because resistance to atypical β-lactam antibiotics (β-lactam antibiotics other than penicillin and cephalosporins) can be mediated by the production of ESBLs and/or AmpCs (40, 41). It has been reported in the literature that ESBLs and AmpCs can hydrolyse carbapenem antibiotics at very low levels (42, 43). This resistance can be horizontally transmitted through environments such as sinks, feed and soil, and the various genetic and transcription factors by which resistance arises are easily activated in the environment (44, 45). The global epidemic of atypical β-lactam-resistant *Enterobacteriaceae* has posed a serious public health threat (44, 46, 47), and the results of this study are worth attention.

In contrast, the detection rate of drug-resistant bacteria in yaks was very low. Yaks live mainly in the western highlands of China and mainly graze in pastures (48). Unlike intensive farming environments, disease is less likely to break out on a large scale and unlike large farms with dedicated veterinarians, highland herders rely on their own experience for the treatment of sick yaks, apart from regular guidance from government veterinarians, and do not have records of drug use or standard prescriptions. This is largely in line with farming in low-income areas (49–51). These areas are more prone to antibiotic abuse compared to larger farms (52). However, because the incidence is low and the use of antibiotics is minimal, drug resistance genes are much less likely to spread.

A US study reported that 39 out of 42 stool samples contained *E. coli* carrying CTX-M (93%) (53). In another German prevalence survey on ESBL-producing *E. coli*, 96.5% of calves in the 10 large rural villages involved were ESBL-positive, 92.9% of which were *E. coli* (54). In contrast, a Nigerian study showed that the CTX-M gene was detected in only 12.8% (32/250) of cattle samples (55). In West and Central Africa, the prevalence of ESBL-*E. coli* in cattle is 0% (56). This explains the higher prevalence in Shandong, a middle- and high-income region, compared to the other regions in this study.

CTX-M-55, CTX-M-79, CTX-M-15, and TEM have been identified not only in animals but also in humans and the environment (57–59). Evidence has shown that antibiotic resistance genes and antibiotic-resistant bacteria can spread to humans *via* contaminated meat, house dust, manure, and waste water discharge (60–62). Meanwhile, direct evidence has shown the environmental transmission of antibiotic resistance genes and their bacterial hosts among livestock and humans (63).

At present, the daily excretion of the dairy cattle manure is as high as 200,000–300,000 t in China. In some places, untreated cattle manure is returned to the field, and resistant bacteria and genes are released into the environment following feces application. Antimicrobial resistance genes can be cotransferred among humans, animals, food, and the environment (64). Bovines are reservoirs and are the active spreaders of ESBL producers. It is necessary to survey and control antibiotic resistance on animal farms.

Our results will help to establish a baseline to guide the interventions in reducing antibiotic resistance in agriculture and on farms. The disadvantages of this study are the sampling area and the limited animal species. To obtain more accurate results, larger samples will be needed.

## Data Availability Statement

The raw data supporting the conclusions of this article will be made available by the authors, without undue reservation.

## Ethics Statement

The animal study was reviewed and approved by Research Ethics Committee of Lanzhou Institute of Husbandry and Pharmaceutical Science of CAAS. Written informed consent was obtained from the owners for the participation of their animals in this study.

## Author Contributions

XW and ZD wrote the manuscript. NL and XW carried out the CTX-M gene detection and the antimicrobial susceptibility testing. WW was responsible for isolation of *E. coli*. LW, BL, XZ, FC, KZ, HC, and HS collected the samples. JZ provided the funding resource and was responsible for the paper quality. All authors contributed to the article and approved the submitted version.

## Funding

This study was supported by the NSCF (grant number 31872520) and China Agriculture Research System of MOF and MARA (grant number CARS-37).

## Conflict of Interest

The authors declare that the research was conducted in the absence of any commercial or financial relationships that could be construed as a potential conflict of interest.

## Publisher's Note

All claims expressed in this article are solely those of the authors and do not necessarily represent those of their affiliated organizations, or those of the publisher, the editors and the reviewers. Any product that may be evaluated in this article, or claim that may be made by its manufacturer, is not guaranteed or endorsed by the publisher.
