# Corrigendum: Prevalence of multidrug-resistant CTX-M extended spectrum beta-lactamase-producing *Escherichia coli* from different bovine faeces in China

**DOI:** 10.3389/fvets.2022.1123496

**Published:** 2023-01-04

**Authors:** Xiaojuan Wei, Weiwei Wang, Ningning Lu, Lingyu Wu, Zhen Dong, Bing Li, Xuzheng Zhou, Fusheng Cheng, Kairen Zhou, Haijian Cheng, Hongmei Shi, Jiyu Zhang

**Affiliations:** ^1^Lanzhou Institute of Husbandry and Pharmaceutical Sciences of Chinese Academy of Agricultural Science (CAAS), Lanzhou, China; ^2^Key Laboratory of New Animal Drug Project of Gansu Province, Lanzhou, China; ^3^Key Laboratory of Veterinary Pharmaceutical Development, Ministry of Agriculture, Lanzhou, China; ^4^Shandong Institute of Animal Science and Veterinary Medicine, Jinan, China; ^5^Gannan Tibetan Autonomous Prefecture Institute of Animal Husbandry Science, Gannan, China

**Keywords:** blaCTX-M, *E. coli*, feces, yak, beef cattle, dairy cattle

In the published article, there was an error in the Funding statement.

This study was supported by the NSCF (grant number 31870520) and China Agriculture Research System of MOF and MARA (grant number CARS-37).

The correct Funding statement appears below.

This study was supported by the NSCF (grant number 31872520) and China Agriculture Research System of MOF and MARA (grant number CARS-37).

The authors apologize for this error and state that this does not change the scientific conclusions of the article in any way. The original article has been updated.

